# Suicide and community justice

**DOI:** 10.1186/s40352-018-0072-7

**Published:** 2018-08-21

**Authors:** Jake Phillips, Nicola Padfield, Loraine Gelsthorpe

**Affiliations:** 1 Department of Law and Criminology, Sheffield Hallam University, Heart of the Campus, Collegiate Crescent, Sheffield, S10 2BQ England; 2 Faculty of Law, University of Cambridge, West Road, CB3 9DZ Cambridge, England; 3 Institute of Criminology, University of Cambridge, Sidgwick Avenue, Cambridge, CB3 9DA England

## Abstract

**Background:**

There has long been concern about the number of people who die in custody in England and Wales, particularly in prisons or police stations. The concern is obviously heightened when people die either at their own hand, or at the hands of others. Yet there has been selective critical gaze, and people who die whilst under probation or community supervision have been neglected (Phillips, J, Gelsthorpe, L, Padfield, N., Criminology & Criminal Justice, 10.1177/1748895817745939, 2017). Given that there is evidence to suggest that contact with the criminal justice system in non-custodial settings is associated with higher mortality rates than those found in the general population, such neglect is concerning.

**Methods:**

This article explores data which has been published since 2016 by Her Majesty’s Prison and Probation Service (HMPPS) on the deaths of offenders whilst under supervision. We draw on data which is collected by probation providers and collated by HMPPS to present original analyses, with particular focus on deaths by suicide. We calculate rates of self-inflicted deaths and rate ratios with the general population and the prison population.

**Results:**

The suicide rates for all groups within the sample are higher than the general population.

**Conclusions:**

We explore the utility of the data in helping us to understand the trends regarding people dying whilst under probation supervision with a particular focus on suicide, and highlight areas where the dataset is deficient. We conclude that whilst the dataset can be used to calculate headline rates of suicide it raises many questions in terms of the extant risks that people on probation face, and we explore ways in which the data can be used more fully to understand this important social and public health issue. We consider ways in which the dataset could be matched with other datasets in future research so that health issues might be brought into the analysis, and reflect on other research methodologies which would add depth to our understanding of why the mortality rate amongst people in contact with the criminal justice system is higher than in the general population.

## Background

This paper explores the under-researched topic of deaths (particularly self-inflicted deaths) of those subject to criminal justice supervision in the community. Whilst in England and Wales there has been significant public (and academic) concern for deaths in custody, the deaths of those subject to community supervision has not been studied nearly as much. This became a cause of concern to us in 2010, when the Howard League for Penal Reform encouraged us to analyse information that they had received from individual Probation Trusts (as they were then): see Gelsthorpe et al. 2012. Since then, more official data has been published. We show in this paper that despite limitations with these official data, there are some important concerns when it comes to suicides of people under probation supervision.

## What do we already know about suicide in community justice settings?

Although the deaths of offenders under supervision have received less attention than deaths in other criminal justice institutions, there is evidence to suggest that the mortality rate amongst this group is higher than the general population. The following section provides a review of existing research on this topic focusing on prevalence, official statistics and risk factors.

### Prevalence

Pritchard et al. (1997) examined suicide and violent death in a six-year cohort of male probationers in England and Wales for the period 1990–1995 and found that males (aged 17–54) had twice the death rate and nine times the suicide rate of the general population. In a study for the Home Office, Sattar (2001) found that deaths following release from prison tended to occur soon after release. A quarter of deaths within her sample of 1267 deaths in the community in England and Wales occurred within 4 weeks of release from prison. Over half occurred within 12 weeks of release, and within 24 weeks of release just under three-quarters of all deaths had occurred. The number of deaths in the community was five times the rate of the 236 deaths of prisoners and the mortality rate for supervisees was four times higher than that for the male general population rate. Solomon and Silvestri (2008) found that the rate of suicide of those under probation supervision was nine times higher than in the general population and higher than in prison. King et al. (2015) found that 13% of suicides in the general population in England and Wales were, or had recently been, under supervision by the criminal justice system. They reported a significantly elevated suicide risk among individuals who had received a police caution, recently been released from prison, recently completed a supervised community sentence, served other community disposals, been remanded as a suspect on police bail and/or dealt with by no further action (King et al., 2015: 175). Interestingly, they found that ‘individuals serving a community sentence under the supervision of the Probation Service had a relatively low risk’ of suicide (2015: 175). King et al.’s (2015: 176) findings were not statistically significant but they point to the potential for probation supervision to serve as a protective factor and ‘as a crucial source of support for vulnerable offenders’.

Internationally, a high mortality rate amongst people under criminal justice supervision in the community has been consistently identified. For example, In Australia, Biles et al. (1999) found that people under probation supervision had a higher mortality rate and suicide rate than the general population and people in prison. In Denmark Webb et al.’s (2011) analysis of suicide amongst people in the criminal justice system identified an elevated risk of suicide and Binswanger et al.’s (2011) analysis of deaths after leaving prison shows a mortality rate that is 3.5 times that of the general population.

### Trends and themes within English and welsh official data

In 2016 the Ministry of Justice (2016a) published, for the first time, data on deaths of offenders in the community. This means that we now have a better understanding of trends regarding deaths within the offender population in England and Wales. That said, it should be noted there are concerns about the quality of these data, as noted elsewhere (Phillips et al. 2017). For example, data from the years 2010–2013 contained many gaps in terms of type of sentence and offence category and it is worth noting that recording practices changed over this period. Moreover, two (out of 21) Community Rehabilitation Companies^1^ (CRCs) failed to report any information by the Government’s cut-off date for the 2016–17 figures (Ministry of Justice 2017a). In order to compensate for this latter point, Government statisticians have adjusted figures for earlier years by removing deaths from the two missing CRCs ‘so as to provide like-for-like comparisons with 2016-17’ (Ministry of Justice 2017a: 5). Thus trends over time should be treated with care.

Nevertheless, the government’s own publication shows that in 2015/2016, there were 725 deaths of offenders in the community, a 30% increase from 557 deaths in the previous year. Of the 725 deaths, 264 were self-inflicted and show an increase of 40% from 2014/2015. A further 68 were accidental and there were 22 apparent homicides. The remaining 371 were from natural causes or unknown (Ministry of Justice, 2017a) Table 1.

**Table 1:** Deaths of offenders in the community by gender and apparent cause, England and Wales (Ministry of Justice 2017a)

	2010/11	2011/12	2012/13	2013/14	2014/15			2015/16			2016/17^a^		
					NPS	CRCs	Total	NPS	CRCs	Total	NPS	CRCs	Total
Males and Females	704	653	634	560	179	379	558	241	497	738	279	469	748
Self-inflicted	148	162	190	189	49	141	190	63	207	270	72	161	233
Natural Causes	238	222	240	214	75	121	196	106	135	241	124	134	258
Homicide	27	28	28	17	5	9	14	6	16	22	13	20	33
Accident	68	49	49	37	13	35	48	21	46	67	14	43	57
Other	31	42	29	25	3	12	15	1	10	11	3	11	14
Unclassified^b^	192	150	98	78	34	61	95	44	83	127	53	100	153

*NPS* National Probation Service; *CRC* Community Rehabilitation Company

^a^Missing returns from 2 CRCs. Caution should be used when comparing with earlier periods

^b^Unclassified deaths refer to those deaths for which a cause was unknown at the time of reporting

The category ‘unclassified’ refers to those deaths for which a cause was unknown *at the time of reporting* and has not yet been updated. The latest data give some information on deaths of offenders supervised by the National Probation Service as opposed to those supervised by the CRC and there are some points worth noting here. As the Ministry of Justice highlights;

In 2016/17, the proportion of self-inflicted deaths in the NPS was lower than the proportion of deaths due to natural causes. The opposite is true of the CRCs, where self-inflicted deaths accounted for a higher proportion of deaths than natural causes. This is only partly explained by the different age distributions of the supervised offenders. When comparing on a like-for- like basis, CRCs had a drop in the number of self-inflicted deaths compared to the previous year, whereas the NPS saw an increase (Ministry of Justice, 2017a: 8).

Drawing again upon the Ministry of Justice’s analysis, there were 372 deaths of offenders under post-release supervision in the community after a custodial sentence in 2016/17. This represented 50% of all deaths of offenders in the community. Although in absolute terms these were similar between NPS and CRCs (180 and 192 deaths respectively), in relative terms deaths under post-release supervision represented 41% of all deaths in the community while supervised by CRCs, but 65% of all deaths while supervised by the NPS. The Ministry of Justice argues that the ‘sizeable increase in deaths since 2014/15, corresponds with the introduction of ORA^2^’. However, it is worth noting that when we break this down to people on post-release supervision, the number of self-inflicted deaths rose from 40 to 117 (an increase of almost 300% between 2013/14 and 2016/17) whilst the number of people on post-release supervision rose by just 179% (from 39,565 to 70,650) over the same period (Ministry of Justice, 2016b). It is possible that changes to recording methods account for some of this discrepancy but probably not all.

It is also worth commenting on deaths that occur within Approved Premises (AP) which are used to house high risk offenders, normally upon release from prison. APs are akin to bail hostels or halfway houses and are generally run and managed by the National Probation Service, although in recent years there has been some outsourcing of certain tasks, such as staffing night cover and there are a number of independent Approved Premises. According to official statistics (Ministry of Justice, 2017a) there were 13 deaths of offenders resident in Approved Premises in 2016/17. The number of deaths have ranged from 9 to 15 deaths a year since 2010/11 which accounts for 2% of all deaths of offenders in the community. The majority of deaths of offenders in Approved Premises were male, while females accounted for three deaths. Approved Premises fit into our current system very uncomfortably not least because there is acute demand for more space. This is particularly the case for women, so much so that a woman won her case before the Supreme Court^3^ in 2017 on the grounds that provision of APs constitutes direct discrimination against women which is unlawful unless justified, and that the Secretary of State had shown no such justification (see *R (Coll) v Secretary of State for Justice* [2017] UKSC 40). At that point there were 94 APs for men, distributed around England and Wales including several in London. There are only 6 APs for women, who constitute 5% of the prison population, and none of them is in London or in Wales. This means that women are much more likely than men to be placed in an AP which is far from their homes and communities. Deaths that occur in Approved Premises are investigated by the Prisons and Probation Ombudsman (PPO) in the same way that deaths that occur in prison are investigated. The people who die whilst residing in an AP are the only probation supervisees whose deaths are investigated by an independent investigatory body in accordance with Article 2 of the Human Rights Act 1998. In our analysis of PPO reports into deaths in APs we found that drug use was a major concern both in terms of the risk associated with problematic drug use that is common to people who are required to live in APs as well as around the poor level of drug treatment available to people in prison in the first place and then during the transition into the community (Phillips et al. 2016). We also raised concerns around staffing levels, especially at weekends, as well as some issues around decision making and partnership working.

In addition to the Ministry of Justice’s analysis we have conducted analysis of the raw data in research on behalf of the Equality and Human Rights Commission which focused on people who died within 28 days of leaving prison. Our analysis (Phillips et al. 2016) showed, confirming findings from elsewhere, that the first week after leaving prison was the highest risk with 66 people dying within 28 days of leaving prison. By far the most common cause of death among this cohort was a self-inflicted overdose (*n* = 44) and the most common offences were acquisitive (*n* = 35), an offence type which is commonly associated with problematic drug use. The majority (*n* = 37) of those 66 deaths occurred in the first 2 weeks after release.

### Risk factors

People under supervision have a higher mortality rate than the general population although data for England and Wales are slightly outdated. However, prevalence only tells part of the story and if concerted action can be taken to prevent suicides in the future we need a better understanding of the risks that people face that may increase the chances of them taking their own lives. Again, the evidence here is relatively weak. That said, drugs and alcohol use features highly amongst people who die in the community (Sattar, 2001; Binswanger et al. 2011). Indeed, research consistently finds that many people who have offended have poor physical and mental health, lead chaotic lifestyles and are more likely to misuse drugs (Mills, 2004; Brooker et al. 2009; Canton, 2008; Singleton et al. 2003; Brooker and Sirdifield, 2013 and Denney et al. 2014). This is important considering mental ill health is one of the main predictors of suicidality (Arsenault-Lapierre, Kim and Turecki, 2004). Writing about the situation in England and Wales, Cook and Borrill (2015: 255) found that the key indicators for an offender to be considered at risk of suicide were previous incidents of self-harm or attempted suicide and, to a lesser extent, ‘coping skills, psychiatric treatment/medication, attitude to self, childhood abuse, current psychological problems/ depression, and history of close relationship problems’. Borrill et al. (2017: 12) analysed the case records of 28 people who died by suicide while under probation supervision in England and highlight ‘the complex association of events and experiences that may contribute towards pathways to suicide among probation service users under supervision’.

More recently still, Mackenzie, Cartwright and Borrill (2018) conducted research with seven probation clients who had attempted suicide. Their participants suggested that bereavement, loss of control over their mental state or situation, difficulties relating to their probation sentence, issues around trusting authorities, and an inability to disclose suicidal feelings were linked to their suicidal feelings and behaviours. Pratt et al. (2006) found that within their sample of 384 suicides that occurred within 12 months of leaving prison main risk factors ‘were increasing age over 25 years, released from a local prison, a history of alcohol misuse or self-harm, a psychiatric diagnosis, and requiring Community Mental Health Services (CMHS) follow-up after release from prison.’

It is clear that previous research has uncovered trends and consistent findings amongst people who die by suicide when under probation supervision. However, as MacKenzie et al. (2013) have argued, there is a need for more research on this important social issue because of the consistent finding that people under supervision are at higher risk of dying by suicide than other populations. In order to update Sattar’s (2001) work and to add some context to our understanding of the suicide rate of people under probation supervision we now turn to new analysis of the data that are collected by Her Majesty’s Prisons and Probation Service (HMPPS) on the number of people who die by suicide when under probation supervision.

## Methods

In the remainder of this article we augment the Ministry of Justice’s analysis of the data on deaths of offenders under supervision to update our knowledge of the suicide rate amongst this population. In order to gain a better understanding of the rate of suicide amongst people under the supervision of probation providers we have conducted new analysis of official data on deaths of offenders under supervision. The data contained in the dataset was collated by HMPPS using forms completed by probation providers when someone dies in accordance with Probation Instruction 01/2014 (Ministry of Justice, 2014). These forms ask for basic demographic data of the offender, cause of death and brief details of what happened in the run up to the death. They are then collated and published in brief by HMPPS as discussed above. We use the data to make comparisons between this group, the general population and people who die by suicide in prison. We have followed the methodology utilised recently by Fazel et al. (2017) to calculate rate ratios between different populations. We have access to the raw data through a data sharing agreement with HMPPS in order to conduct research on behalf of the Equality and Human Rights Commission, and use this as the basis for our analysis because of the decision by HMPPS to amend their published data as a result of the non-submission of data from two CRCs as mentioned above.

In order to make comparisons with other populations we have calculated the suicide rate amongst people under supervision per 100,000. We have used the number of people on the caseload available from the Ministry of Justice (2016b) as this bears the strongest resemblance to measures used in both Fazel et al.’s (2017) study, Sattar’s (2001) research and suicide rates from the Office for National Statistics (Office for National Statistics, 2017).

There are limitations to this approach. Firstly, there is a definitional issue. Ministry of Justice data on the deaths of offenders under supervision includes ‘category of death’. Within this category there is a label of ‘self-inflicted’. However, this is not necessarily the same definition as used by the ONS nor the same definition of suicide used by the prison service. Moreover, in many cases the cause of death recorded by probation providers is ‘Apparent - on the basis of information received’ rather than having been confirmed by a coroner’s inquest verdict or death certificate. That said, this is all we have. Secondly, our use of the number of people on the caseload as a means with which to calculate suicide rates may result in inaccurate data. We are using the caseload as a proxy measure for the annual average population as described by Sattar (2001). This allows for the comparison between three groups in question: offenders in the community, people in prison, and the general population.

## Suicide rates and rate ratios of offenders who die when in the community

To make comparisons with suicide rates in other contexts we have calculated the suicide rate amongst people under probation supervision using the total number self-inflicted deaths between 2010/11 and 2015/16 and calculated a mean suicide rate with 95% confidence intervals. This allows us to compare the suicide rate of offenders in the community with the suicide of people in prison, as calculated by Fazel et al. (2017). Table 2 shows that the suicide rate of offenders, regardless of age or gender, is both higher than that in prison (by a factor of 1.42) and the general population (by a factor of 8.67). This reflects findings from other research looking at similar populations (Sattar 2001).

**Table 2:** Suicide rate and rate ratio of people dying by suicide under supervision, in prison and in the general population

Total number of suicides of offenders under supervision (2010/11–2015/16)	Annual suicide rate (offenders in community) / 100,000		Annual suicide rate (prisoners) /100,000 (Fazel et al. 2017)		Annual suicide rate (general population) /100,000 aged 30–49 (ONS)	Rate ratios		
	Rate	95% CI	Rate	95% CI		Supervision/prison suicide rate ratio	Supervision/general population rate ratio	Prison/general population rate ratio (Fazel et al. 2017)
1619	118	99–137	83	66–100	13.6	1.42	8.67	6.1

### Gender and suicide

Suicide rates are strongly correlated with gender with death rates from suicide being four-to-five times higher for men than for women across the European Union (OECD, 2018). Thus we have calculated suicide rates and rate ratios for men and women separately. Table 3 shows that the suicide rate for men under supervision in the community is 6 times higher than the general population whilst the rate ratio between men in prison and the general population is 3.9. Meanwhile, the rate ratio between women under probation supervision and the general population is 29.2 compared with a rate ratio between women in prison and the general population of 8.9. The increased risk of suicide for women in prison has long been recognised (Sandler and Coles, 2018) and our analysis suggests that the risk for women offenders in the community is even higher.

**Table 3:** Suicide rate of people under probation supervision compared with suicide rate of people in prison and in general population (2010/11–2015/16)

Gender	Suicide rate per 100,000 offenders under supervision (2015–16)		Annual suicide rate Gen pop suicide rate/100,000 aged 30–49	Rate ratios	
	Rate	95% CI		Supervision/general population rate ratio	Prison/general population rate ratio (Fazel et al. 2017)
Men	105	95.2–115	18.8	5.6	3.9
Women	146	115–175	5	29.2	8.9

### Age and suicide

Age is strongly correlated with suicide, with men aged 40 to 44 having the highest age-specific suicide rate at 15.3 per 100,000 (Office for National Statistics, 2017). Tables 3 and 4 shows the age-specific rates for people under supervision compared with the equivalent age-specific rates in the general population.

**Table 4:** Age-specific suicide rates for men under probation supervision, 2015/16

Age band^a^	Number of deaths by suicide by people under probation supervision (2015–16)	Age specific suicide rate/100000 on caseload	Age specific suicide rate in general population/100,000	Rate ratio
21–24	21	214.61	14.8	14.5
25–29	28	238.70	17.5	13.6
30–39	86	527.41	19	27.8
40–49	66	650.05	23.5	27.7
50–59	19	449.49	21	21.4

^a^Age groups 18–20 and 60+ have been removed due to low numbers

Table 4 shows that the suicide rate for all people under probation supervision is higher than the general population. The rate ratio between men aged 30–49 is the highest with a RR of 28. In Table 5 we see that women also present a higher a risk of suicide when under probation supervision with particularly high rate ratios being present amongst women aged 30–39. However, these are small numbers and so must be treated with caution.

**Table 5:** Age-specific suicide rates for women under probation supervision, 2015–16

Age band^a^	Number of deaths by suicide by people under probation supervision (2015–16)	Age specific suicide rate/100000 on caseload	Age specific suicide rate in general population/100,000	Rate ratio
18–29	9	212.01	4.2	50.48
30–39	14	414.45	4.8	86.34
40–49	10	468.82	6.8	68.94

^a^Age groups 18–20, 21–24 and 25–29 have been combined due to low numbers and age groups 50–59 and 60+ have been removed due to low numbers

### Suicide rate according to sentence type

In order to ascertain a link between sentence type and suicide rate we have calculated the suicide rate amongst men and women on different sentences.

Table 6 shows the suicide rate broken down by gender and sentence type. It is not possible to break this down by age group due to small numbers. It is worth noting, however, that suicide rates are higher than the general population across all sentences, and that women serving a post-release sentence appear to face a particularly high risk, as also highlighted by Sandler and Coles (2018).

**Table 6:** Suicide rate amongst people under probation supervision by sentence type

Suicide rate/100000 on caseload (2015/16)	Community Order	Suspended Sentence Order	License/post-release
Men	142.69	103.27	128.59
Women	156.28	86.51	206.53

### Ethnicity

The actual numbers of people dying by suicide when broken down by ethnicity are too small for meaningful analysis of suicide rates broken down by ethnicity. Moreover, the Office for National Statistics does not collect suicide rates for ethnic groups and so rate ratios with the general population cannot be calculated. However, BAME groups are over-represented in the criminal justice system so this does require some analysis.

Thus, we have compared the proportion of suicides that occur amongst different ethnic groups with the proportion of people in those ethnic groups under supervision. There is no data available for the breakdown of people on license/post-release supervision and so this only refers to people on a Community Order or Suspended Sentence Order. Table 7 shows that white men account for 97% of all suicides but only 81% of the probation caseload, suggesting that they are at higher risk than other groups. All the women who died by suicide in 2015/16 on a Community Order or Suspended Sentence Order were White despite this group making up 88% of the caseload. Despite being based on small numbers this reflects Pratt et al. (2006) finding that non-white ethnicity is a protective factor.

**Table 7:** Proportion of suicides completed by people under supervision according to ethnicity compared with proportion of caseload belonging to ethnic group, 2015–16

Ethnic group	Proportion of all suicides amongst probationers completed by ethnic group (%)	Proportion of probation caseload belonging to ethnic group (%)
Asian	1.5	7
Black	1.5	7
Mixed	0.7	3
White	97	81
Unknown	2	2

### Temporal trends in the risk of suicide

**Fig. 1:**
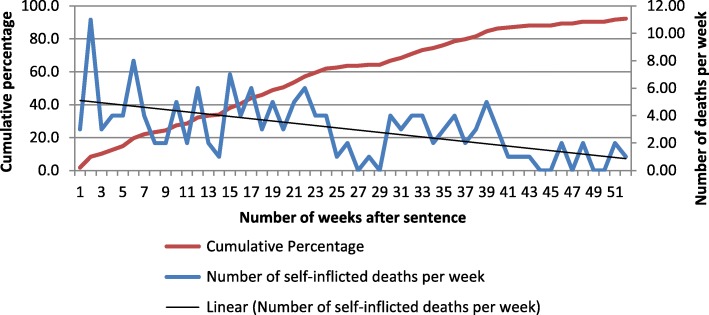
Number of deaths per week after sentence and cumulative percentage of self-inflicted deaths, 2015–16

As discussed previously, research by ourselves (Phillips et al. 2016) and others (Pratt et al., 2006) suggests there is an increased risk of suicide and other mortality soon after release from prison. In order to ascertain whether a similar increased risk exists for people after being sentenced to a community sanction (both Community Orders and Suspended Sentence Orders) we calculated the time period after sentence that a death occurred, again using 2015/16 data. Figure 1 shows that the number of deaths per week after sentence decreases slightly over a period of a year suggesting that there does appear to be a slightly increased risk of suicide in the first weeks after sentence.

### Day of the week

**Fig. 2:**
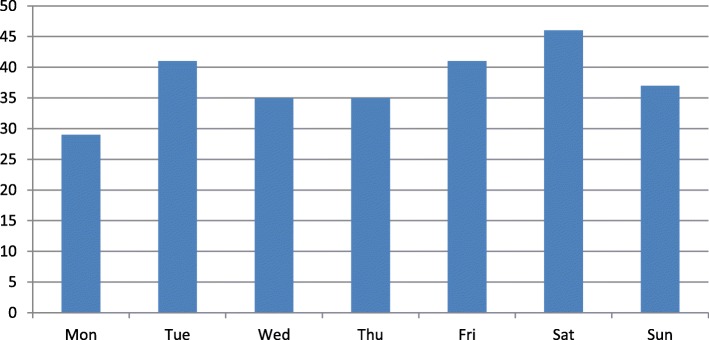
Number of deaths per day of the week, 2015–16

For interest, we are including a table which shows the number of deaths per day of the week. We used the date of death entered on the form completed by the offender manager to calculate the number of deaths that occurred on each day of the week for the whole of 2015/16. There are no clear differences here, except for the apparent increase towards the end of the week. We note that Tuesday shows a high number of deaths, but there is no apparent reason for this. We are left with a number of questions as to whether this is a ‘benefits’ day, or the day on which rent is due, or whether it is simply a day when the harsh realities of the week set in. There could be many reasons for this Fig. 2.

## Discussion

Our analysis demonstrates that the suicide rate amongst people under probation supervision, including those serving a Community Order, Suspended Sentence Order or on licence/post-release supervision, is higher than the general population. Our review of the literature highlights some of the reasons for this disparity. English law and practice is characterised by its complexity, and by frequency of change. In the last 40 years governments (of every political persuasion) have regularly changed the rules, policy and practice relating to sentences, especially those related to the supervision of offenders in the community. This means that the collection of relevant data will have been affected by changes to policy, recording practices, the advent and abolition of organisations most notably the recent part-privatisation of probation which sees different organisations (a mixture of private public) being responsible for offenders who pose different risks. The implications of this are that the data collated by Her Majesty’s Prison and Probation Service are unlikely to paint a complete picture (Phillips et al. 2017). One hundred and twenty seven of the 725 deaths that were recorded in 2015/16 are classified as ‘Unknown’ and in only 88 of the 264 self-inflicted deaths recorded in 2015/16 was the cause of death verifiable via an inquest verdict or death certificate. It is likely that self-inflicted deaths will have been under-recorded because of this. This is the most significant limitation of this analysis: that some deaths will not have been recorded at all, and that others will have been recorded wrongly. Moreover, we have included self-inflicted deaths by drug overdose in our analysis as it is not always known whether there was intent to kill oneself in these circumstances which may have inflated the rate. The decision to include these deaths was made on the basis of Fazel et al.’s (2017) study showing that the majority of countries do not require intent to be proven for a classification of suicide to be recorded in statistics on deaths in prison thus enabling comparison with their analyses.

We have been unable to calculate suicide rates for different ethnic groups, nor would we have been able to compare these with the general population due to a lack of data from ONS. Moreover, the data do not allow for calculating suicide rates according to offence because of a lack of detail in the dataset (for example, 74 of the self-inflicted deaths in 2015/16 are listed as either ‘Other Summary Offence’ or ‘Other Indictable Offence’). As argued by Fazel et al. (2017) this is an area for future research as it is possible that the heterogeneity of the caseload leads to a dilution of high-risk groups. These are all important areas for future investigation but are contingent upon data being available. That said, we have calculated suicide rates for people on different sentences which might, at least, be used as a proxy measure for risk of harm if not risk of reoffending.

In spite of these limitations, these data provide an updated understanding of the suicide rate amongst people on probation. The suicide rate amongst people under probation supervision is clearly higher than that of the general population and, it seems, of people in prison. However, they do little to highlight the issues which may explain the high level of mortality observed. Whether this is to do with the process of being under supervision is not clear.

Whilst quantitative data on the numbers of deaths provides useful headline figures in regard to prevalence and general risk it does little to shed light on the underlying causes of a self-inflicted death. We cannot tell which of the risk factors identified above are most prevalent with this group, nor does it tell us what has been happening in an offender’s life prior to them dying by suicide. Thus there is scope for making greater use of these data. Firstly, it creates the potential for data matching to make the most of other data held by probation providers and other government departments. For example, Delius, the case management system that is in use by all probation providers in England and Wales should be able to provide information on the extent of an offender’s engagement with their sentence in the run up to a suicide attempt. Through the use of a specific code at the expiration of a sentence it should become possible to identify who dies by suicide and then conduct analysis on their patterns of engagement. Analysis of this kind could augment Borrill et al.’s (2017) findings that missed appointments appear to be correlated with subsequent suicide. Similarly, OASys assessments and OGRS scores would enable a much greater understanding of how risk of reoffending and harm interacts with the risk of suicide. Other health data, as collected by primary or secondary healthcare providers might also shed light on the extent to which risk factors correlated with suicide in the general population, such as mental ill health, hold up within this specific population.

Secondly, there is much more scope for collecting data around the life stories of many of these people who died. One approach would be to adopt that taken by Borrill et al. (2017) as mentioned above, who looked at the cases of 28 service users who took their own lives in one large probation area between 2010 and 2013. They highlight the need for further awareness-raising amongst frontline probation staff of sharing crucial information; something that would be missed with high-level analysis of Delius records. An alternative would be to conduct interviews with people who have attempted suicide whilst on probation supervision, as per Mackenzie et al. (2018), or to speak with family members who have had a relative die whilst under supervision in the community. This would reflect the methodology adopted by the PPO following a death in custody or the EHRC when conducting their inquiry into the deaths of adults with mental ill health in custodial settings (EHRC, 2015). This methodological approach would allow for the collection of data which adds considerable depth and nuance to the necessarily abstract picture presented above. These additional forms of research would add to our understanding of the risk of suicide at an individual level which, currently, HMPPS data is unable to address.

However, it is likely there are ecological factors at play which influence the risk of suicide amongst people on probation. At this stage it is important to lay out the context in England and Wales. When an offender is released from prison, or receives a community order, they will be subject to supervision. Radical change was introduced in 2014–15, when a significant part of the previously publically funded Probation Trusts was privatised. Since the Offender Rehabilitation Act 2014 came into force on 1 February 2015 the number of offenders on post-release supervision has soared. Privately run Community Rehabilitation Companies now carry out the majority of probation work working with low and medium risk offenders whilst a newly created National Probation Service supervises high risk offenders. It was intended that CRCs would take around 70% of the work, leaving with NPS with 30% of the caseload to supervise. In reality the split is nearer 60:40. This has resulted in the NPS being overstretched whilst CRCs have received less income than anticipated and thus have struggled to provide an adequate level of service (National Audit Office, 2016; HM Inspectorate of Probation, 2017).

This is the context in which we must understand the deaths of people serving sentence supervised by an increasingly fragmented system. In addition to the structural change to probation providers, it is important to note that the prison system in England and Wales is undergoing a turbulent time. HM Inspectorate of Prisons reports have consistently pointed to increased levels of drug use, a high incidence of mental ill health and low staffing levels (HM Inspectorate of Prisons, 2017). There has been a 20% increase in violence inside prisons in England and Wales (Ministry of Justice, 2017b). When considering the effect that this may have on people on post-release supervision it raises the possibility of these prison based issues influencing the risk of people dying by suicide upon release.

There is a real gap in provision at the point of release. There have been several negative reports on Through the Gate (TTG) services provided by CRCs (HM Inspectorate of Probation, 2016, 2017; Taylor et al., 2017) whilst Padfield’s (2017a, 2017b) research into parole underscores the absence of suitable provision to which prisoners can be released. A joint inspection by the HM Inspectorates of Probation and Prisons found that 15% of prisoners leave prison without accommodation to go to and reported that they ‘did not see any innovative work by CRCs to make access to accommodation easier’ (HM Inspectorate of Probation, 2016: 22). Prisons have acknowledged that they are sending people out with a tent and sleeping bag or a ‘cold weather’ mountaineering blanket (HM Inspectorate of Prisons, 2016). Moreover, the 2017 Annual Report of the Chief Inspector of Probation (HM Inspectorate of Probation, 2017) highlighted the increasing number of supervision sessions carried out with offenders on the telephone which will necessarily limit the potential for good quality assessment and support. Whilst our data cannot say whether these gaps in service provision upon release have a direct impact upon the suicide rate it is an area which requires further investigation through analysis of specific cases.

Healthcare is a key issue when it comes to the risk of suicide and subsequent prevention. As already discussed, people on probation have greater physical and mental health needs when compared with the general population (Brooker et al. 2012). This becomes ever more important when we consider the fact that the same research shows that ‘offenders’ engagement with health services was incommensurate with their likely level of need’ (Brooker et al. 2009: 45). What’s more, research into the commissioning of healthcare for people under probation supervision shows that ‘only 12 per cent of Mental Health Trusts provided a service to support approved premises and just 32 per cent provided clinics in probation’ (Brooker et al. 2015). The provision of healthcare to people serving a community sanction is clearly inadequate and may well contribute to the high suicide rate amongst this population.

Offenders in the community cannot, and should not, be subject to the same level of supervision as those in prison. Criminal justice staff will have less ability to intervene and prevent some deaths. Elsewhere, we have made the case for an ‘ethic of care’ (Phillips et al. 2017). It is interesting to note that the official statistics comment that

Offenders under supervision in the community (other than, to an extent, those occurring in approved premises) are not in the care of NOMS in the way they are when in custody. The influence probation officers have on offenders, in terms of their health and well-being, cannot be compared meaningfully to the influence staff working in prison have in relation to deaths in custody. (Ministry of Justice 2017a: 3)

Whilst it may well be true that the ability of staff to influence a person on the brink of suicide is much more limited in the community, we would urge the authorities to accept that they have responsibility here too. Given what is known about the vulnerabilities of many people under community supervision, much more could be done to support them. Hence the need for a much stronger ‘ethic of care’.

Our final point is about justice. There is an emerging body of evidence which demonstrates that there are a distinct set of pains associated with being on probation (Hayes 2018). Indeed, McNeill has characterised penal supervision as a Malopticon which works to degrade rather than construct positive identities: ‘in the Malopticon penal subjects suffer … the pain of *not* being seen; at least not as they would recognize themselves’ (2018: 19, emphasis in original). We would argue that the lack of attention paid to deaths in the community in terms of poor data, lack of independent investigation, inadequate healthcare provision and a less obvious duty of care is one of the most significant forms of the civic degradation which, for McNeill (2018), is part and parcel of being under penal supervision. That many of these deaths appear to be either ‘missing, ignored or unimportant’ raises questions about the ways in which the state achieves justice for offenders, victims and the communities in which they reside.

## Conclusion

This article has drawn on existing literature to show that the suicide rate amongst people under statutory probation supervision is higher than the general population. The article has also highlighted some of the key risk factors which might explain this relatively high mortality rate. We have then conducted new analysis on national data that were collected and collated by HMPPS on the number of people who have died by suicide by when under probation supervision. Despite the limitations in the data there is a clear finding that the suicide rate across all groups under supervision is higher than the general population. Women appear to be at higher risk, relatively speaking, than men. This, we argue, may be the result of a range of factors related to the system of probation supervision and healthcare provision in England and Wales. We have also pointed to some potential avenues for future research which take both quantitative and qualitative approaches. It is only a mixed approach which will enable a better understanding of the risk factors within different groups. Once suicides by people under probation supervision receive the attention they deserve then practitioners and policymakers will be able to implement policies which serve to reduce the suicide rate amongst this already vulnerable group.

## Footnotes

1 These are private probation providers that were introduced following the implementation of the Offender Rehabilitation Act 2014. For more on the potential implications of these reforms, see below.

2 Offender Rehabilitation Act 2014: the Act of Parliament which allowed the division of probation services into privately managed companies alongside the National Probation Service: see below.

3 The Supreme Court is the final court of appeal in the UK for civil cases, and for criminal cases from England, Wales and Northern Ireland.
